# What do people need to know about endocrine disrupting chemicals and health? A mental models approach using focus groups of community-engaged research teams and a national survey

**DOI:** 10.1186/s12889-025-25561-4

**Published:** 2025-11-22

**Authors:** Katherine E. Boronow, Julia Green Brody

**Affiliations:** Silent Spring Institute, 320 Nevada Street, Suite 302, Newton, MA 02460 USA

**Keywords:** Endocrine disrupting chemicals, Environmental health literacy, Mental models, Risk communication

## Abstract

**Background:**

Endocrine disrupting chemicals (EDCs), which interfere with the body’s natural hormones, are ubiquitous in everyday environments and consumer products. Nearly everyone is routinely exposed, and growing evidence links them to adverse health outcomes including cancers, impaired fertility, metabolic disorders, and neurodevelopmental effects. Major medical and scientific groups recommend exposure reduction. To make informed decisions about individual- and societal-level exposures to EDCs, people need relevant knowledge. Knowledge is one component of environmental health literacy, a multidimensional concept supporting readiness to protect health from environmental risks. This study sought to develop expert consensus about communications targets for EDCs and to learn how public knowledge matches these targets.

**Methods:**

We convened focus groups with community-engaged research teams (*n* = 38) to define targets for public understanding. We coded transcripts, mapped causal pathways influencing EDC exposures and health outcomes using a mental models approach, and identified communication priorities. We then fielded a quantitative online survey among adults living in the U.S. (*n* = 504) to compare their knowledge with the mental model. We computed response frequencies and used multiple regression to evaluate associations between a knowledge index and participant characteristics.

**Results:**

Focus group participants highlighted that people need to know that EDCs affect nearly all systems in the human body and that scientific evidence supports limiting exposure. They emphasized that policy controls can be more effective than personal action at reducing exposure, and that current U.S. chemicals regulations are not protective. Survey respondents were generally aware that EDCs can affect fertility, cancer, and child brain development (84–90%, *n* = 426–452), and they had some understanding of exposure pathways (58–86%, *n* = 295–435). However, most participants had large knowledge gaps about U.S. chemicals regulation and wrongly believed that chemicals must be safety-tested before being used in products (82%, *n* = 414), that product ingredients must be disclosed (73%, *n* = 368), and that restricted chemicals cannot be replaced by similar substitutes (63%, *n* = 317).

**Conclusions:**

U.S. adults typically understood that EDCs affect health. However, incomplete information about how people get exposed to EDCs and misconceptions about U.S. chemicals regulations limit appropriate actions. These knowledge gaps are targets for future communications about EDCs and harmful chemicals more broadly.

**Supplementary Information:**

The online version contains supplementary material available at 10.1186/s12889-025-25561-4.

## Introduction

Rapidly evolving science has identified chemicals in everyday environments and consumer products that act as endocrine disrupting compounds (EDCs)—chemicals that mimic or disrupt natural hormone signaling. Common examples include current-use chemicals like bisphenols, phthalates, and per- and polyfluoroalkyl substances (PFAS) and banned persistent chemicals like polybrominated diphenyl ethers (PBDEs), polychlorinated biphenyls (PCBs), and dichlorodiphenyltrichloroethane (DDT) [1], and hundreds of less studied chemicals also display endocrine disrupting activity [2]. EDCs are used in pesticides, flame retardants, plastics, personal care products, food packaging, and waterproof and stain-resistant coatings. These chemicals are used commercially at large scale: in 2019 (the most recent year available), aggregated U.S. production volume for just six phthalates restricted in children’s products (di-(2-ethylhexyl) phthalate, diisobutyl phthalate, dibutyl phthalate, dicyclohexyl phthalate, diisononyl phthalate, and benzyl butyl phthalate) was estimated between 62.9–181 million pounds, while the volume of bisphenol A alone was estimated between 1–5 billion pounds [3]. Complex mixtures of EDCs are routinely detected in people’s bodies and homes [4, 5].

Major medical and scientific groups agree there is sufficient evidence of harmful health effects to recommend exposure reduction. Expert assessments include statements by the Endocrine Society, International Federation of Gynecology and Obstetrics, American Academy of Pediatrics, American Heart Association, World Health Organization and United Nations Environment Programme, and consensus workgroups [1, 6–11]. Authoritative reviews support links between EDCs and obesity, metabolic disorders like diabetes, male and female reproductive health, hormonal cancers, thyroid function, and neurodevelopment [1, 12, 13]. The annual cost of healthcare and lost wages from EDCs was estimated at $340 billion in the U.S. in 2010 [14].

Importantly, studies demonstrate that exposures vary with product choices and individuals can reduce exposure for some compounds by changing behaviors [15–18]. In addition, as consumers and citizens, people can influence corporate practices, legislation, and agency rulemaking. For example, California revised its flammability standards in response to public awareness about flame retardants [19], and activists played a key role pushing states to establish enforceable Maximum Contaminant Levels (MCLs) for PFAS in drinking water [20].

However, people first need to understand how EDCs affect health and the factors that influence exposure before they can decide to reduce risks [21, 22]. To facilitate these decisions, public health messages must prioritize critical information, build on what people already know, and correct misconceptions [22]. We drew from two theoretical frameworks—mental models approaches and the emerging field of environmental health literacy (EHL)—to generate empirical data to inform EDC communications.

A mental model integrates knowledge and beliefs into a causal story that forms a basis for decisions. Influence diagrams are a visual representation of how components of the mental model interact and are formed by deconstructing a hazard into set of causal pathways. Mental models research seeks to identify differences between the mental models held by lay people and those held by experts as a basis for risk communication [22]. The typical research approach begins by characterizing expert understanding about a hazard by engaging with subject matter experts before assessing public understanding. Assessing lay understanding often integrates qualitative approaches such as interviews (for depth and richness) as well as quantitative approaches such as surveys (for high-level understanding and prevalence), although each method can be applied in isolation. The mental models approach has been applied to environmental exposures such as dioxin, radon, and electromagnetic fields [23–25], and to other urgent environmental health threats like climate change [26]. Although not a mental models approach, one study prepared, a priori, the authors’ causal schematic of EDC exposure [27].

Similar to mental models, EHL encompasses people’s understanding of relationships between environmental exposures and health and their ability to take action to protect health [21, 28, 29]. Like health literacy, EHL is expected to influence individual and population health, but unlike health literacy, it operates in a community context. Because environmental hazards rarely impact one person at a time, EHL engages explicitly with community or systems-level solutions. Another defining feature is the emphasis on translating knowledge into action [29].

Blending these two frameworks, we sought to create a functional mental model for environmental health literacy about endocrine disruptors (EDC-EHL) that supports informed individual action and integrates societal forces. Our functional mental model still organizes information about EDC exposures and health according to causal pathways, but it omits some aspects of a complete expert mental model because it is limited to what lay people need to know for decision-making. Understanding discrepancies between people’s beliefs and the functional mental model is critical to intervening on public understanding [30].

Existing data suggest strengths and weaknesses in public knowledge about EDCs. In a quantitative study among 135 women, age 49–56 years, most participants had basic knowledge about how people are exposed to EDCs and about their health effects [28]. For example, 92% knew that babies in the womb can be exposed to harmful chemicals during pregnancy, and 93% or more identified brain development, fertility, and cancer as health risks associated with the chemicals in the study (PBDEs, PFAS, PCBs, and legacy pesticides). However, participants also held two major misconceptions: 76% incorrectly thought that chemicals in the U.S. are safety tested before being used in products, and 70% believed that doctors could tell how blood levels of EDCs would affect future health [28]. Similarly, in interviews with EDC biomonitoring participants, a common theme was learning that chemicals in consumer products are not thoroughly tested for safety and few chemicals have health-based exposure guidelines [31]. Studies from Canada, France, Turkey, and Northern Ireland found limited awareness of EDCs and their sources [32–35]. One study that focused only on understanding about phthalates found that, among 117 postpartum people, 69–74% correctly agreed with each of three statements measuring “general phthalate knowledge,” including that phthalates are found in personal care products and can harm health [36].

In this study, we convened focus groups with researchers who study EDCs, community partners, outreach and communications professionals, and clinicians to develop a mental model for what the public needs to know about this emerging public health threat. Then, based on this mental model, we developed a structured survey to assess EDC-EHL in a large geographically and socioeconomically diverse population. These results provide a systematic look at what people living in the U.S. know—and don’t know—about how EDCs can affect health and the individual and social controls mediating exposure. Findings will support the development of communications that promote public health by enabling people to make informed decisions about harmful chemical exposures in their homes and communities.

## Methods

**Fig. 1:**
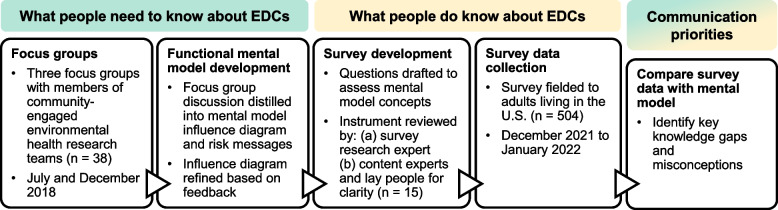
Overview of the study design and methodological approach

This study was conducted in two phases (Fig. 1). First, we held focus groups to identify what people need to know about EDCs. Second, we fielded a survey to learn what people actually know about EDCs. By synthesizing results from both phases, we identified communication priorities for EDC risk messages. This study was approved by the WCG Institutional Review Board.

### Focus groups and mental model development

To develop an expert mental model for EDC-EHL, we recruited researchers and community partners from teams funded by the National Institute of Environmental Health Sciences and National Cancer Institute. We recruited attendees of the July 2018 Integration Meeting of the Breast Cancer and the Environment Research Program (BCERP) and December 2018 Partnerships for Environmental Public Health (PEPH) Annual Meeting. We targeted these meetings because the BCERP teams and many PEPH members conduct research on EDCs and because of the strong emphasis on involving community partners and research translation. Researchers and community partners who attend these meetings are working in environmental health research, and they have interest and experience in public communications about science and contacts with community members to inform their thinking about science communication.

Focus groups were conducted in person and participants (*n* = 38) provided informed consent. The moderator guide (see Supplemental Information) was organized around what people need to know about six topics to have functional EDC-EHL, including: defining EDCs, sources of exposure, biological action, health effects, reducing exposure, and misconceptions about EDCs. Sessions lasted approximately 75 to 90 min and were audio-recorded and subsequently transcribed. Participants who were not federal employees were offered a $50 Amazon.com gift card and two participants were randomly selected to win a Detox Me Action Kit (valued at $399), which tested for EDCs in urine.

Both authors independently coded all transcripts in Dedoose and periodically discussed coding definitions to achieve consistency. Initial codes were based on the moderator guide, and additional codes were created based on themes in the transcripts and discussion between the coders. For each code we summarized associated excerpts into main points. We then synthesized these main points in two ways. We constructed an influence diagram that organizes key concepts about EDC exposures and health according to causal pathways elucidated in the focus groups (i.e., the functional mental model). The influence diagram was reviewed for clarity by environmental health colleagues and presented at scientific conferences. We also distilled key themes for public messaging to improve environmental health literacy, and these themes provide important context for the influence diagram.

### Survey development and analysis

We developed a survey instrument to measure knowledge, attitudes, and behaviors related to EDC exposures and health. Here, we report on the knowledge questions (see Supplemental Information) because they directly measure elements of the EDC-EHL mental model. Responses were required for all questions. Participants answered 34 true–false statements using response categories of true, probably true, probably false, and false. A response set that includes ‘probably true’ and ‘probably false’ is commonly used in mental model studies because this allows for an assessment of “lean” or confidence [22]. Participants answered yes, no, or ‘I don’t know’ to 10 questions about the presence of EDCs in common household items. They answered yes or no to three questions assessing awareness of specific chemicals (bisphenol A [BPA], parabens, and per- and polyfluoroalkyl substances [PFAS]). For each chemical, participants who responded yes were then asked to choose one of four statements describing different exposure sources or ‘I don’t know.’ Due to a technical error, our data contained some missing responses and zero ‘I don’t know’ responses for these three follow-up questions about chemical group sources. Out of caution, we report them as ‘no response.’

Questions were selected to provide coverage of content domains represented by the mental model. Some of the questions were adapted from a survey previously fielded by the authors in a study assessing the effects of reporting back to participants on their personal blood level results for 42 EDCs [28]. Additionally, we developed new questions based on our subject matter expertise. The draft survey instrument was reviewed multiple times by an expert in survey research (M. Induni) to ensure clarity and conformance with best practices, such as avoiding leading questions that suggest a desirable answer and double-barreled questions, which incorporate more than one topic. Faulty questions were reworded. We used snowball sampling to solicit feedback on the clarity, scientific accuracy, and completeness of the draft survey from contacts within our network, including those with and without subject matter expertise. Reviewers (*n* = 15) provided open-ended feedback for each question and the survey overall, and this feedback was used to eliminate or revise questions that were unclear or had multiple interpretations. We attempted to balance true and false statements, with the final instrument having 18 true statements and 16 false statements.

Participants were recruited through Qualtrics Research Services and completed online informed consent and eligibility questions. Eligible participants were adults aged 18–80 years currently living in the United States, and quotas were applied to ensure representation across gender, race and ethnicity, geographic, and educational groups. We identified low quality responses and excluded participants if they: provided the same response to all questions in the knowledge or community action sections, gave nonsensical open-ended responses, or completed the survey in less than 160 s. We did not collect information on people who started but did not complete the survey. To reduce bias due to order effects, question order was randomized within the true–false and household-items knowledge components. After completing the survey, participants were provided explanations about the correct answers to the knowledge questions. Participants who completed the survey received compensation that had a monetary value between $2.10 and $2.80. Survey responses were collected from December 20, 2021 to January 20, 2022.

Race and ethnicity was coded as Hispanic/Latinx if participants responded yes to having Hispanic, Latino/a, or Spanish origin; otherwise, race and ethnicity was coded according to participants’ selected race, or as multiracial or other if they selected more than one race or provided an alternative category. Current state of residence was categorized into U.S. Census Bureau regions. Knowledge responses were re-coded as correct or incorrect based on the correct answer for each question. Because the correct answer varies by question, re-coding was necessary to create a variable that can be intuitively interpreted for all questions in the same way. Responses of “probably true” and “probably false” were re-coded as “correct but unsure” and “incorrect but unsure.” We computed response frequencies for all questions. We summed correct and correct but unsure responses to the true–false questions to calculate a knowledge index. We used multiple regression to evaluate mutually-adjusted associations between the knowledge index and participant characteristics (gender, education, race and ethnicity, age, having children in the household, and household income). Statistical analysis was conducted in R (version 4.3.1; R Development Core Team).

Finally, we assessed correspondence between public knowledge, as revealed by the survey results, and the functional mental model and themes for public messaging derived from the focus groups.

## Results

We first describe the EDC-EHL functional mental model derived from expert focus groups and then present survey results about what U.S. residents know about EDCs.

### Focus groups and mental model development

We conducted two focus groups with BCERP affiliates (*n* = 10 and 9, held July 10 and 12, 2018, respectively) and one focus group with attendees of the PEPH Annual Meeting (*n* = 19, held December 13, 2018). The focus groups included researchers in toxicology and epidemiology, research staff doing community outreach and education, community members engaged with EDC research, social scientists, doctors and nurses, and communications experts. Attendees reflected the composition of the overall meetings, with greater representation by research scientists at BCERP and by outreach and education staff at PEPH. Among the 38 participants, 31 were non-Hispanic White, 6 were Hispanic/Latinx, and 1 was non-Hispanic Black; 4 were men.

**Fig. 2:**
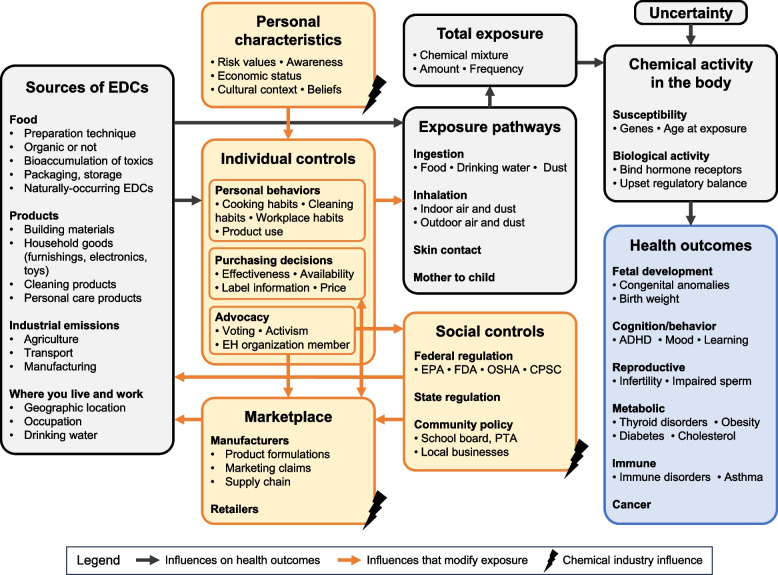
Influence diagram constructed from expert focus groups depicting the mental model for functional environmental health literacy about endocrine disrupting chemicals. Node contents list key components (in bold) and representative examples (bullet points). The primary endpoint of the model, health outcomes, is highlighted in blue. Grey nodes and arrows show the direct pathway from EDC sources to health outcomes. Orange nodes and arrows are influences that modify exposure levels along the direct pathway

Results of our thematic analysis are presented in two ways. Figure 2 depicts the functional mental model as an influence diagram, and Table 1 summarizes key messages and discussion points.

**Table 1:** Key messages to improve environmental health literacy about endocrine-disrupting chemicals (EDC-EHL), derived from thematic analysis of the focus groups

Domain	Key message	Discussion points^a^
Sources of EDCs	EDCs are found in many consumer products, living environments, and foods that people encounter every day	• Nearly everyone is exposed to EDCs in their daily lives • Highlighting salient exposure sources for an audience can help limit information overload: “If you’re talking to farm workers, you’re definitely gonna be talking about pesticides. If you’re talking to folks in a nail salon, you’re gonna be talking about…personal care products.”
	Except in contamination scenarios, people do not need to learn specific chemical names to be able to make decisions about EDCs	• Complicated chemical names can be “overwhelming” and “off-putting.” • Learning chemical names can limit what people try to avoid and leave them vulnerable to newer replacements • Some participants thought that naming specific chemicals can be empowering, including in consumer exposure contexts
Chemical Activity in the Body	EDCs dysregulate the body’s fundamental processes	• Knowing how hormones normally operate in the body is critical to understanding the disrupting activity of some environmental chemicals. Participants conceptualized the actions of EDCs on normal hormone activity in three ways: o EDCs bind to hormone receptors (as in the classic lock-and-key metaphor) and either stimulate the receptor inappropriately or prevent the natural hormone from binding o EDCs alter the message that hormones are sending from one part of the body to another, or prevent it from being delivered: “In a sense that if I want to send a message to you but it’s the telephone tag game where I whisper something…and it gets distorted, and in a way, maybe endocrine disruptors are distorting the message.” o EDCs disturb the entire body’s regulatory balance—influencing not just reproduction, which is most-often associated with hormones, but also growth, metabolism, behavior, and more—by causing the body’s complex and carefully tuned systems to become unbalanced or disordered: “And those instructions [from hormones] can lay the blueprint during development for changes that will be permanent and then they also, during adulthood, modulate things on a daily, hourly, monthly basis. And that whole system, we have chemicals in the environment that actually interfere with that communication.”
	EDCs pose greater risk to health during periods of rapid development, such as exposure in the womb	• EDCs have greater health effect during “windows of susceptibility” including fetal development, childhood development, puberty, pregnancy, and breastfeeding • Exposure in utero or during childhood can affect development with lifelong consequences, including increased risk of disease later in life • Genetic background and health conditions can also affect susceptibility to EDCs
Total Exposure	Exposures to EDCs can add up to pose a risk to health	• Scientific findings support concern about both lower and higher doses of EDCs • Specific exposure levels associated with health outcomes are not known for most EDCs, and identifying these harmful levels for individual chemicals is not helpful for characterizing cumulative risk • Risk may accumulate from frequent exposures to many EDCs over time: “Yeah, I try to tell people similar things like this exposure to one of these chemicals is not gonna cause your breast cancer. It’s not a necessary or sufficient cause, or at least we don’t know that it is. But, exposure to it could potentially increase your risks slightly… the idea of all these little risks adding up.”
Health Outcomes	EDC exposures contribute to diverse health outcomes affecting nearly all systems in the human body	• Health impacts of EDCs extend beyond female reproductive disorders traditionally associated with hormones and long latency diseases such as cancer • EDCs can affect individuals without manifesting as a condition requiring medical treatment • Valuable evidence for health outcomes can come from well-designed animal studies or from epidemiological (human) studies • Human studies often include participants with especially high exposures, such as from a contamination event or occupational exposure • Unlike human studies, animal studies can be precisely controlled, and animal testing for pharmaceutical safety is well-accepted: “If a drug that we’re developing for people kills animals, we never would give it to a person. So, if we can use them [animals] to test safety, then we should use them to test safety.” • Relying on animal studies can be problematic if the studies were not designed to fully capture EDC effects, for example, omitting environmentally relevant doses or relying on crude endpoints instead of sensitive markers of health
Individual Controls	Personal actions can reduce some exposures to EDCs	• Personal choices can lower certain exposures, and, for EDCs with short half-lives, actions can have rapid effects on levels in the body • Telling people about the efficacy of exposure reduction actions can be motivating: “…that sort of gives them a hope that you can do something about this, that okay, right now they probably have to hear about bad stuff in our bodies, but if we little-by-little substitute certain things then we can reduce harm.” • When exposure reduction is easy or low-cost, there are few downsides to precaution, even if the benefit is uncertain. Consumers may be able to identify some EDC-containing products that don’t offer many benefits
	Individual action has limitations and is insufficient to address EDC exposure	• Cost can be a barrier to personal actions like changing products or eating organic • Safer alternatives are not always sold in local stores and some groups have less access to online retail (such as people in rural areas, the elderly, or underbanked households) • The U.S. regulatory system creates barriers to informed purchasing decisions (see *Social controls*) • Chemical exposures from where a person lives can be difficult for individuals to address on their own—for example, industrial or agricultural contamination of private drinking water wells or conditions in rental or public housing—and moving may not be possible
Marketplace	Consumer purchasing decisions and advocacy can create market shift away from EDCs	• Decreasing consumer demand for EDC-containing products can drive manufacturer changes, such as reformulating products or introducing alternatives • Consumer impacts are magnified if retailers and organizational buyers avoid EDCs • Advocacy campaigns (such as efforts to remove methylene chloride paint strippers) and business-government partnerships (like the San Francisco Healthy Nail Salon program) can influence companies
Social controls	The U.S. system of chemicals regulation does not protect people from EDC exposures	• Gaps in the U.S. regulatory system leave people exposed to EDCs, and correcting misconceptions about U.S. chemicals regulation is a key target for EDC-EHL • Chemicals regulation in the U.S. is highly fragmented across agencies, including the EPA, Food and Drug Administration (FDA), Consumer Product Safety Commission (CPSC), and Occupational Safety and Health Administration (OSHA), which makes it harder for people to understand the existing regulatory framework as well as how best to target policy change • The U.S. does not require testing chemicals for safety before they are used in products. Chemicals or products are regulated only after a problem is observed, a system participants described as “backwards.” In contrast, the European Union takes a more precautionary approach that includes limited testing of chemicals for safety before use • Awareness of limited safety testing is key for public understanding. Participants repeatedly cited the common misconception, “If it’s sold in the store, it must be safe.” This misconception reflects incorrect beliefs that all products sold in the U.S. are regulated by the government for safety or that all chemicals are tested for safety before they are allowed in products • The U.S. regulatory system allows potentially unsafe substitution, which occurs when manufacturers replace one chemical that was banned or voluntarily removed because of consumer scrutiny with a structurally similar replacement that is likely to have similar toxicity • Many types of consumer products have no federal requirement to label ingredients, and requirements for cosmetics and personal care products are incomplete, for example, permitting use of the catchall term “fragrance.” • Manufacturers take advantage of gaps in required ingredient disclosure—and gaps in consumer EHL—by prominently advertising chemicals that are *not* used in their products (e.g., BPA-free, PFOA-free) without also highlighting what chemicals were substituted in their place: “*Speaker 1*: So, if you think of going to the store…and it says, BPA-free! You may not know what that means, but that sounds like it's good. They took something bad out *Speaker 2*: They might have put something bad in…We [focus group participants] know how that works. But, the public doesn’t.” • Drinking water is not comprehensively regulated to limit exposure to EDCs. Private wells are essentially unregulated and are often contaminated from nearby commercial activity. Although municipal water supplies must comply with EPA regulations, few EDCs are covered
	Public policy can lower EDC exposures for everyone	• Public policy can produce greater public health impact than individual behavior change, and advocacy is important for changing public policy. People influence public policy by voicing their opinion to and educating elected representatives, and by voting: “I think people need to know that their voting choices can make a difference for their health and well-being.” • Policy change has been effective at the state level. For example, California and New York have passed legislation increasing ingredient disclosure for household cleaning products, and advocacy by PFAS-affected communities is effectively targeting state health advisories and regulatory levels • People can engage locally, for example, through municipal government, school boards and parent-teacher organizations, and grassroots community organizations
Uncertainty	Evidence for harm from EDC exposures is sufficient to warrant a precautionary approach toward exposure reduction	• Despite scientific uncertainty (for example, about what dose will cause harm, or mapping the mechanistic pathway from exposure to disease), the strength of evidence from many lines of study supports reducing exposure to EDCs now based on the precautionary principle • Evidence comes from both animal and epidemiological studies, and well-designed animal studies should be considered credible and relevant • Waiting for scientific “proof” will lead to unnecessary and prolonged exposure in the general population: “If we wait till we prove that X, Y, and Z is harming us, well we’re gonna get through numerous generations. They [chemical manufacturers] love to do that argument about, ‘Well, you can’t prove it. You can’t prove it.’ And so, I think we have to pull back and focus in on that precautionary principle.” • If people are informed about the evidence, they can make decisions based on their own risk tolerance
	The chemical industry strategically deploys misinformation to create uncertainty	• Chemical manufacturers sabotage scientific and public discourse about EDCs by funding and disseminating biased research, using strategies previously deployed by the tobacco industry: “I think one thing that's worth talking about is the fact that there’s this whole other world of the manufacturers who produce these chemicals, who fight what we do, who produce data to suggest that we're wrong, who manufacture doubt.”

^a^Quoted excerpts from focus group participants are used to illustrate themes discussed by the group

The influence diagram shows the pathway from EDC sources to health outcomes and maps influences that modify exposure along the pathway (Fig. 2). Each node lists essential components (in bold) and selected representative examples (as bullet points), but the influence diagram is not an exhaustive inventory of focus group discussion. The diagram starts with the diversity of sources of EDCs, including from food, consumer products, industry, and the workplace. Exposure to these sources is mediated by personal choices, decisions by manufacturers and retailers, and public policies. Personal characteristics such as risk awareness and risk values, economic position, and cultural context influence the many behaviors that affect exposures, including both personal habits and civic participation. The chemical industry also shapes peoples’ choices by influencing beliefs about EDCs, regulatory policies, and the availability of marketplace alternatives, which in turn affects sources and exposure. After exposure to EDCs, the unique mixture and dose experienced by each person interacts with their biological susceptibility, including timing of exposure and genetics. While recognizing uncertainty, the influence pathways culminate in the health outcomes node, which captures scientific evidence for the effects of EDCs on fetal development, cognition and behavior, reproduction, metabolic health, immune function, and cancers.

The influence diagram provides an overarching relational framework, and within this framework we used thematic analysis to define core priorities for EDC-EHL communications. Themes covered 8 domains encompassing where EDCs come from, how they affect the body, what level of EDC exposure matters, how individuals can limit exposures, how regulatory and market forces can affect exposures, and the role of uncertainty. Thematic analysis results are presented in Table 1, and brief highlights follow.

Focus group participants identified that communication priorities for EDC-EHL can be setting-specific, particularly in populations with distinct exposure concerns, for example, from occupational exposures or local industrial contamination. For ubiquitous exposures from daily living, most participants thought that learning chemical names was not necessary or helpful. To explain the biological impacts of EDCs, they emphasized the role of hormones in controlling many critical processes throughout the body, not just reproduction, and that EDCs disrupt the body’s sensitive balance of systems. Windows of susceptibility in the life cycle when exposures are more harmful were considered important.

Focus group participants discussed how to motivate and inform exposure reduction. Hopeful messages about the efficacy of small actions were encouraged, while at the same time, participants cautioned communicators to be mindful of barriers to individual actions such as cost and access. Themes also addressed how personal decisions can interact with systems solutions: consumer demand can drive marketplace changes, while civic engagement can impact local, state, and national policy.

Communicating about the role of public policy was considered vital to empower the most effective strategies for exposure reduction and mitigate frustration about barriers to individual behavior change. Messages to explain U.S. regulatory gaps, specifically that chemicals do not have to be tested before they are used in products, were identified as high priorities. Other issues included lax requirements for ingredient disclosure and misleading marketing claims (e.g., BPA-free), and industry research designed to create doubt. To counter industry misinformation about chemical safety, focus groups participants discussed the types of evidence that scientists use to understand EDC health effects, including well-designed animal and human studies, and the costs to health of waiting for “proof.”

### Survey results

Results of our survey of EDC-EHL in a diverse sample of people living in the U.S. allowed us to compare public understanding with the mental model and related themes. We received 670 complete responses and excluded 166 responses based on our exclusion criteria, resulting in a final sample of 504 participants. Demographic characteristics are summarized in Table 2. Median time to complete the survey was 9.8 min (IQR: 7.1–14.4 min).

**Table 2:** Characteristics of survey participants (*n* = 504)

Characteristic	Response level	Number (%)
Gender	Male	237 (47)
	Female	266 (53)
	Nonbinary	1 (0)
Race and ethnicity	Non-Hispanic White	309 (61)
	Non-Hispanic Black or African American	70 (14)
	Non-Hispanic Asian	26 (5)
	Non-Hispanic American Indian or Alaska Native	2 (0)
	Non-Hispanic multiracial or other	8 (2)
	Hispanic/Latinx	89 (18)
Highest education	Up to high school graduate/GED	121 (24)
	Some college	138 (27)
	Technical school, trade school, or associate degree	65 (13)
	Bachelor's degree or higher	180 (36)
Total household income	Less than $35,000	189 (38)
	$35,000 to less than $50,000	83 (16)
	$50,000 to less than $75,000	115 (23)
	More than $75,000	117 (23)
Age group	18–24	62 (12)
	25–44	157 (31)
	45–64	134 (27)
	65–80	151 (30)
Children under 18 in the household	Yes	138 (27)
	No	366 (73)
Born in the U.S	Yes	480 (95)
	No	24 (5)
Census region	Northeast	88 (17)
	Midwest	106 (21)
	South	203 (40)
	West	107 (21)

**Fig. 3:**
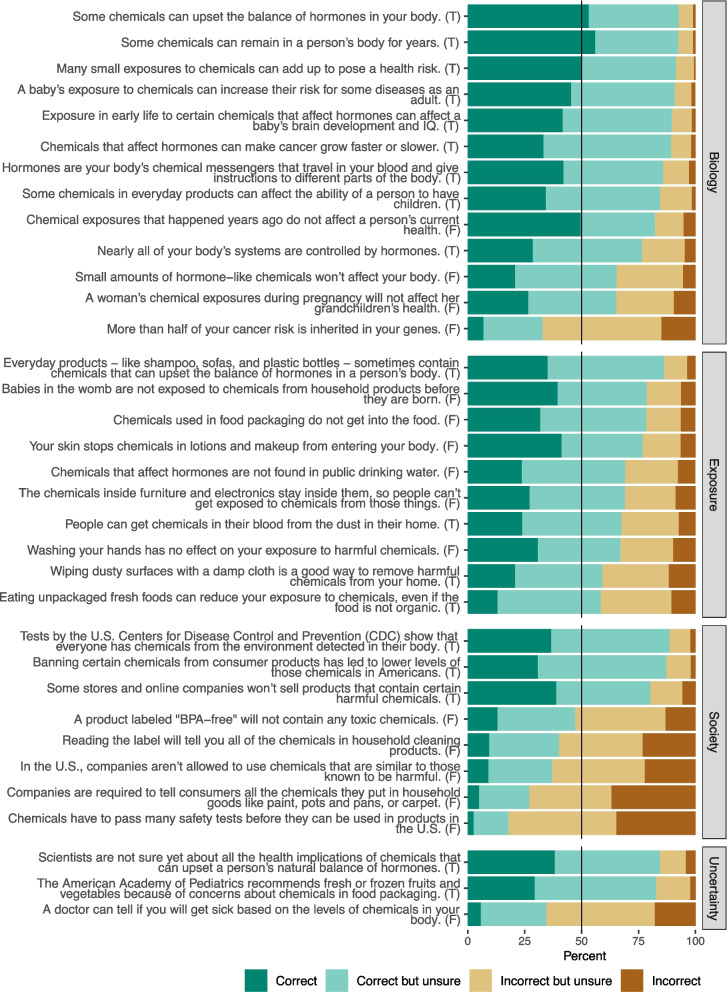
Distribution of responses for each true–false knowledge question (*n* = 504). Questions are grouped by mental model domain (gray labels) and ordered by descending percent correct (includes correct and correct but unsure). The correct answer to each question is shown at the end of the question text (T = true, F = false). Summary data are available in Table S1

Rates of correct responses to the true–false questions are summarized in Fig. 3 and Table S1. For ease of interpretation, we consolidated questions into four overarching domains: biology, exposure, society, and uncertainty. Of 34 questions, 15 (44%) had a rate of correct responses (combining correct and correct but unsure) above 80%, and 9 of these were in the biology domain. Participants generally understood basic concepts that chemicals found in everyday products can interfere with hormones and that these chemicals can affect health (including brain development, fertility, and cancer).

Participants had somewhat less robust understanding overall of how people are exposed to EDCs, with correct rates ranging from 58 to 86% in the exposure domain. However, participants performed the poorest in the society domain: of the 7 questions scoring below 50% correct, 5 were in this domain and addressed safety testing, potentially unsafe substitutions, and labeling of chemical ingredients in the U.S.

The total number of correct responses per participant for the 34 mental model questions ranged from 11–33, with a median of 25 (IQR = 20–27). Female gender, education beyond high school, and age over 45 years were associated with a higher knowledge index in a multiple regression model (Table 3). Non-Hispanic Black and Hispanic race and ethnicity and having children living at home were associated with a lower number of correct responses (Table 3). There was no association with household income (Table 3).

**Table 3:** Multiple regression model evaluating associations between demographic variables and environmental health knowledge index (*n* = 504)

Variable	Level	Estimate (95% CI)	*P*-value
Intercept	Intercept	22.8 (21.3, 24.3)	< 0.001
Gender	Male	ref	ref
	Female	0.9 (0.2, 1.7)	0.013
	Nonbinary	NR	NR
Education	Up to high school graduate/GED	ref	ref
	Some college	1.2 (0.2, 2.2)	0.016
	Technical school, trade school, or associate degree	1.2 (0, 2.5)	0.054
	Bachelor's degree or higher	1.5 (0.5, 2.6)	0.004
Race and ethnicity	Non-Hispanic White	ref	ref
	Non-Hispanic Black or African American	−2.1 (−3.2, −1)	< 0.001
	Non-Hispanic Asian	−1.5 (−3.1, 0.2)	0.084
	Non-Hispanic American Indian or Alaska Native	NR	NR
	Non-Hispanic multiracial or other	NR	NR
	Hispanic/Latinx	−1.8 (−2.8, −0.7)	0.001
Children under 18 in the household	No	ref	ref
	Yes	−1.4 (−2.3, −0.5)	0.003
Total household income	Less than $35,000	ref	ref
	$35,000 to less than $50,000	−0.7 (−1.8, 0.3)	0.18
	$50,000 to less than $75,000	−0.8 (−1.8, 0.2)	0.1
	More than $75,000	0.1 (−0.9, 1.1)	0.81
Age	18–24	ref	ref
	25–44	−0.4 (−1.7, 0.8)	0.5
	45–64	1.4 (0.1, 2.8)	0.035
	65–80	1.7 (0.3, 3.1)	0.014

*CI* confidence interval, *ref* reference level, *NR* not reported (for cells containing fewer than 10 observations)

**Fig. 4:**
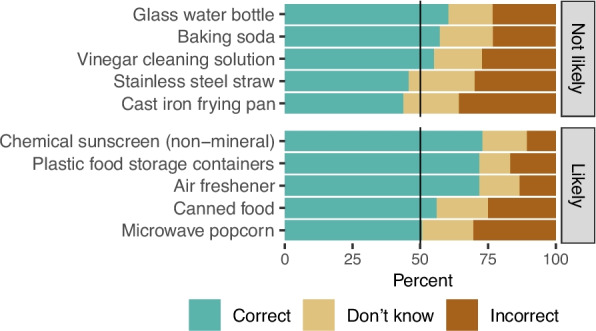
Distribution of responses for each household item question (*n* = 504). Questions are grouped by source type (likely or not likely to contain EDCs) and ordered by descending percent correct. Summary data are available in Table S2

**Fig. 5:**
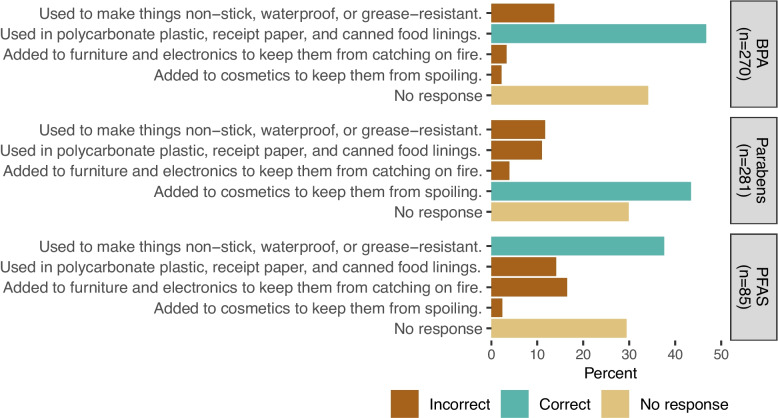
Distribution of responses for each chemical group source question, among participants who responded that they had heard of the chemical before. Summary data are available in Table S3

Participants were not skilled at discerning which household items were likely to contain EDCs. The correct rate for each item ranged from 44 to 73% (Fig. 4, Table S2), and rates tended to be higher for items likely to contain EDCs. We also asked participants about their familiarity with three types of chemicals: BPA, PFAS, and parabens. Slightly over half of participants said they had heard of BPA (54%) and parabens (56%), whereas only 17% had heard of PFAS. Among people who had heard of each chemical, fewer than half correctly identified its function (38% to 47%) (Fig. 5, Table S3).

### Correspondence between expert risk messages and public knowledge

Here we examine how the survey results (Figs. 3–5) relate to themes for public messaging identified by the focus groups (shown in italics below and Table 1).

*EDCs are found in many consumer products, living environments, and foods that people encounter every day.* 86% of survey participants knew that everyday consumer products sometimes contain chemicals that affect hormones. However, when tasked with identifying items likely to contain EDCs, participants did poorly. Three items with the highest correct rates were chemical sunscreen (non-mineral) (73%), plastic food storage containers (72%), and air freshener (72%). When asked about ways that people are exposed, 77–79% of participants knew that people can be exposed to chemicals through contact with their skin and that chemicals can migrate from food packaging into food. Somewhat fewer knew that house dust is an exposure source (68%) or that chemicals in furnishings and electronics are released into the indoor environment (69%). Another important and under-recognized exposure pathway was public drinking water (69%).

*Except in contamination scenarios, people do not need to learn specific chemical names to be able to make decisions about EDCs.* Awareness of chemical group names was low overall, and awareness of a chemical group did not reliably translate to knowing where those chemicals are found. This is consistent with the focus group finding that teaching chemical names was not a communications priority.

*Exposures to EDCs can add up to pose a risk to health.* The survey group demonstrated familiarity with a cumulative risk model, with 92% of participants stating that many small exposures to chemicals can add up to pose a health risk. Fewer participants (65%) understood that EDCs are biologically active at low levels and that small amounts of hormone-like chemicals can affect the body. Participants were widely aware that some chemicals can persist for years in a person’s body (92%) and that chemicals from the environment have been found in nearly everyone (89%).

*EDCs dysregulate the body’s fundamental processes.* Many people (86%) had baseline understanding that hormones are chemical messengers that travel throughout the body, and nearly everyone (93%) stated that some chemicals can upset the balance of hormones. Fewer people (77%) understood that hormones control nearly all the body’s systems, which is a key concept underpinning why EDCs can have widespread impacts on health.

*EDCs pose greater risk to health during periods of rapid development, such as exposure in the womb.* Survey participants did well on related questions: 91% thought that early-life exposures can affect adult disease risk and 82% said that chemical exposures from years ago can affect current health. While 79% understood that fetuses are exposed to chemicals from household products during pregnancy, fewer people (65%) were aware that chemical exposures during pregnancy could also impact grandchildren.

*EDC exposures contribute to diverse health outcomes affecting nearly all systems in the human body.* Participants knew that chemical exposures can affect children’s brain development (90%) and adult fertility (84%), and they knew that chemicals that affect hormones affect cancer growth (89%). However, expressing a major misconception about cancer risk, 67% incorrectly responded that more than half of a person’s cancer risk is attributable to inherited genes. Participants also had the most uncertainty about this question, with 78% of participants falling into the “unsure” categories.

*Personal actions can reduce some exposures to EDCs.* Fewer than two-thirds of participants recognized simple steps to reduce chemical exposures, including washing hands (67%), wiping dusty surfaces with a damp cloth (59%), and eating unpackaged fresh food even if it is not organic (58%). Fewer than half (47%) knew that the marketing language “BPA-free” did not guarantee the absence of harmful chemicals.

*Consumer purchasing decisions and advocacy can create market shift away from EDCs.* 80% of participants knew that some companies restrict certain harmful chemicals from their products.

*The U.S. system of chemicals regulation does not protect people from EDC exposures.* Participants had very limited knowledge of U.S. chemicals regulation: only 18% knew that chemicals do not have to be safety tested before being used in products, and 37% knew that there are not limits on replacing restricted chemicals with potentially unsafe substitutes. Only 27% knew that ingredient disclosure is not required for many types of household products (like carpet or cookware), and slightly more (40%) knew that it is not required for household cleaning products.

*Public policy can lower EDC exposures for everyone.* Despite holding many misconceptions about U.S. chemicals regulation, participants did understand the efficacy of public policy, with 87% agreeing that banning certain chemicals from consumer products has led to lower exposures to those chemicals.

*Evidence for harm from EDC exposures is sufficient to warrant a precautionary approach toward exposure reduction.* Most participants (83%) correctly thought that an authoritative medical society, the American Academy of Pediatrics, recommends limiting exposure to chemicals in food packaging. However, a substantial fraction (65%) believed that doctors can predict health harms based on a person’s chemical levels, although this overstates current knowledge of EDCs. Still, most participants recognized uncertainty in what is known about EDCs, with 84% responding that scientists are not sure about all the health implications of chemicals that affect hormones.

## Discussion

Through focus group discussions with researchers, outreach staff, and community partners, we produced a mental model of functional environmental health literacy about EDCs and tested this model against the knowledge and beliefs of a general sample of U.S. residents. The multilevel EDC-EHL mental model defines core biological and societal processes that influence health outcomes from EDC exposures. Experts emphasized the need for people to understand that EDCs disrupt sensitive systems that control nearly all aspects of health and well-being, and that current U.S. policies do not protect against these exposures for many consumer product chemicals. Yet, in a survey of U.S residents, we found that most people did not understand key societal factors that affect exposure to EDCs, such as lack of safety testing before chemicals are allowed in products. These knowledge gaps limit people’s ability to make informed decisions and represent an important target for risk communication. Notably, our results are relevant to other environmental toxicants that share, in part, biological processes, exposure scenarios, and regulatory context. Correcting misconceptions can change how people evaluate risks and impact personal purchasing decisions and engagement with systems-level solutions.

As expected, the functional mental model for EDC-EHL is consistent with the scientific literature but distilled to higher-level messages important for public decision-making. For example, whether EDCs exhibit non-monotonic dose–response relationships—where lower doses can have different and perhaps stronger effects than higher doses—is debated by academic researchers, industry scientists, and regulators because of the implications for how EDCs are regulated for health risks [37], but our experts did not elevate this as a key message. Rather, they offered a cumulative risk model as a way for the public to understand the harms of ongoing exposures to mixtures of EDCs at levels experienced in daily life, and the survey results suggested that this model was intuitive to lay people. Additionally, the focus groups discussed epidemiological and animal evidence for health effects of EDCs but not new approach methods (NAMs) like in vitro and in silico testing that have gained momentum more recently [38]. As NAMs are validated, they may become a more important source of evidence in the future, and as the body of research on EDCs grows, updates to the functional mental model may be needed. However, we do not think that expert understanding of the foundational science about EDCs has changed since the focus groups were conducted in 2018. Indeed, the Endocrine Society’s 2025 position statement on EDCs reaffirms core scientific consensus from their 2015 scientific statement, and many of their current positions speak to themes identified in our focus groups, including the importance of cumulative and mixture effects and recommendations for ensuring protective regulation of EDCs [1, 39]. Although the functional model developed by our study will include information that is familiar to any scientist who studies EDCs, it is innovative because it represents consensus about key targets for EDC communications targets, and, by clarifying the components of EDC-EHL, it becomes possible to intervene on specific elements of public understanding based on our survey results.

A strength of our focus group approach was recruiting research teams practicing community-engaged research who were experts in the science of EDCs and committed to translational outcomes. Our 38 focus group participants also included many varied forms of expertise (e.g., toxicologists, social scientists, communication specialists), which supports the completeness of the consensus model. We generally observed agreement for the primary themes, even though participants had ample opportunity to add points that had not been mentioned. One exception was different opinions about when people benefit from knowing specific chemical names. Holding focus groups at relevant conferences enabled us to convene large numbers of geographically-dispersed attendees in person; a limitation is that we did not include anyone who was unable to attend. Previous research has shown that 20 responses are sufficient to reach saturation of new ideas using a mental model approach [22], and our focus groups included nearly twice that many.

In comparison with the expert mental model, survey data from U.S. residents revealed damaging misconceptions and knowledge gaps. Most survey participants had limited ability to identify sources of EDCs and recognize effective interventions on exposures, which directly limits their ability to take personal protective action. Further, they had even less knowledge of chemicals regulation, including the lack of requirements for safety testing and ingredient disclosure, which critically undermines people’s *motivation* to act. The functional mental model highlights how individual action and systems-level change are interacting domains: public policy and marketplace actors influence how people encounter EDCs (i.e., from what sources, at what levels), and individuals can respond through advocacy and by using purchasing decisions to spur market shift. However, misconceptions about societal controls disrupt these feedback loops: for example, if people wrongly believe that chemicals are strictly regulated or that product labelling is fully transparent, then their decisions are based on false premises.

Consider that about half of participants wrongly believed that a product labeled “BPA-free” would not contain any toxic chemicals. In reality, “BPA-free” products could include structurally similar substitutes or unstudied chemicals. These claims are often a form of greenwashing, in which products are made to look more environmentally friendly than they are, and research shows that such claims bias consumer preferences [40]. Chemical-free claims exploit consumer naivete about ingredient disclosure and use of similar substitutes, as seen in our results. In 2021, California highlighted this misleading practice when it banned advertising claims like “BPA-free” on cookware packaging when other chemicals in the same class are added [41], but such claims remain acceptable for other product types and markets.

Our results are consistent with results from an overlapping subset of survey questions fielded in 2015 to 135 women [28]. In both studies, knowledge about safety testing for chemicals and what doctors can predict based on chemical levels were major misconceptions, whereas knowledge about health outcomes was stronger. The consistency of findings across the two studies supports their generalizability and that knowledge of EDCs was relatively stable over the period the studies were conducted. In this study we further differentiated “certain” from “unsure” responses. Large proportions of uncertain responses—both incorrect and correct and across all domains—suggests most people would benefit from broad education about EDCs, consistent with other studies reporting incomplete knowledge and lower levels of awareness about EDCs [32–35]. More detailed comparison is possible for awareness of two specific chemical classes, BPA and parabens. Compared to two studies of reproductive-age women in Canada and Turkey, participants in our study (including both women and men) were more likely to have heard of BPA (54% compared to 34–38%) but less likely to have heard of parabens (56% compared to 72–78%) [34, 35]. In focus groups of men and women in Northern Ireland, the “majority” of participants were familiar with BPA and this was in part due encountering BPA-free products [32].

The current study population was bigger, more diverse in gender, age, and geographic location, and included greater representation of Hispanic and Asian populations than our prior research. This enabled us to examine demographic associations with EDC-EHL. We found small positive associations with age greater than 45 years, education past high school, and female identity, and small negative associations with non-Hispanic Black, non-Hispanic Asian, and Hispanic race and ethnicities (compared to non-Hispanic White race and ethnicity) and with having children living in the home. Some EDC exposures are disproportionately higher among Hispanic and non-Hispanic Black women compared to non-Hispanic White women [42], so targeting culturally appropriate EDC-EHL communications specifically for these audiences is a priority. Families with children are another important audience, because childhood exposures can impact lifelong disease course. To further target communications to specific audiences, future research could probe how knowledge varies by demographic group across specific mental model domains and how other social identities—such as political affiliation or personal health history (e.g., prior cancer diagnosis)—interact with beliefs about EDCs. Future research may also incorporate methods that complement our mental model approach, such as cultural consensus approaches, which focus on the level of agreement in beliefs within groups of experts or lay people [43].

The ability to measure EHL in specific topic areas, such as EDCs, is a prerequisite to tracking how population-level EHL changes over time and evaluating the effectiveness of interventions. Our study contributes by identifying key knowledge components of EDC-EHL and provides a snapshot of public understanding. A strength of our quantitative survey approach is that it allowed us to assess the population prevalence of components of EDC-EHL, but a tradeoff is that this method does not capture the public mental model as richly as open-ended interviews could, and future work will benefit from qualitatively examining public understanding and perceptions. Additionally, although we attempted to mitigate common sources of survey bias, for example, by randomizing question order within sections and balancing true and false statements, some bias may persist. In particular, earlier questions may have increased awareness that EDCs are widespread and subsequently prompted participants to overestimate their presence in household items. A further limitation is that we focus on EDC knowledge, knowing that this is only one component of EHL, and knowledge alone is insufficient to influence health behaviors [44]. Further, we aimed to cover core concepts shared across diverse types of EDCs, and additional approaches are needed for specific classes of chemicals and audiences. For example, Tomsho et al. measured EHL about phthalates among parents of young children, which necessitated questions tailored to the specifics of that chemical class [36].

As results of our study are applied to EDC communications, we expect that correcting misunderstandings about social controls will change how people perceive risks posed by everyday chemicals, which can, in turn, impact decisions about what to buy and how to engage with community, state, or national policy. Some EDC researchers and relevant professional societies are already engaged in critiquing current policies and proposing solutions [9, 39, 45–47], but the audiences for these articles are other academics and policy makers. Alongside these expert communications, it is critical to translate key messages about U.S. chemicals regulation for the public. Because social controls for EDCs are relevant to all types of harmful chemical exposures, these messages can also be integrated into general communications about environmental chemicals. Based on our results, we make the following recommendations for how to improve communications about EDCs for the public:

*Address evidence that lowering EDC exposure can reduce health risk.* The likelihood of adopting EDC-lowering behaviors depends, in part, on the perceived benefits of the behavior [44]. We identified two barriers to people’s understanding of the health benefits of exposure reduction. First, most survey participants incorrectly believed that more than half of cancer risk comes from inherited genes. This misconception underplays the role of environmental risk factors including EDCs in cancer development and, importantly, undermines perceived effectiveness of risk-reducing behaviors. Second, our focus groups emphasized the importance of promoting a precautionary approach based on both laboratory and human evidence, yet a commentary from a major cancer conference noted that health communications are often dismissive of evidence from molecular and animal studies [48]. Instead, communications should convey the full range of evidence and highlight the breadth of health outcomes associated with EDCs. Focus group experts emphasized telling people about the incremental benefits of even small steps toward exposure reduction.

*Directly address misconceptions about regulatory controls and provide guidance for taking individual and collective action.* Current risk communications about EDCs focus on personal actions for reducing exposure from consumer products rather than on the underlying systems that enable exposures. Examples include the Breast Cancer and the Environment Research Program (BCERP) education materials for parents and caregivers, American Academy of Pediatrics guidance to families, and Western States Pediatric Environmental Health Specialty Unit (PEHSU) prescriptions for prevention, one example of a toolkit supported by the Prescriptions (Rx) for Prevention program [9, 49–51]. These materials are valuable to support informed exposure reduction now, and new materials can target other EDC exposures and audiences. Additionally, we encourage future communications to address systems-level components of the EDC-EHL mental model, such as highlighting regulatory gaps and connecting individuals to opportunities for collective action. The Toxic Matters website from the UCSF Program on Reproductive Health and the Environment has addressed certain aspects of this by including three ways to engage with government in their guide to reducing chemical exposures [52]. In another study, peripartum people expressed receptivity to information about both individual- and community-level exposure reduction, and they asked researchers to prioritize recommendations to help them efficiently allocate their limited mental and financial resources for this issue [53].

*Leverage communication techniques tested to combat misinformation.* The focus groups identified how industry uses tactics to distort people’s EDC-EHL and prevent regulation. These tactics have been detailed elsewhere for EDCs specifically [54–56] and as a broader playbook deployed repeatedly for issues like tobacco, fossil fuels, and lead [57, 58]. To counteract misinformation, communications about EDCs should draw from lessons learned, for example, from climate change communications, and deploy tested strategies such as inoculation and using culturally appropriate messengers to deliver and affirm relevant knowledge [59]. The survey results suggest that doctors may already be considered authoritative sources of information about EDCs and therefore able to serve as trusted messengers. Because most doctors have limited training on environmental chemicals including EDCs [60, 61], building knowledge among doctors and nurses represents a critical steppingstone towards public EDC-EHL. Pediatricians and obstetricians may be especially receptive to integrating recommendations about EDCs into preventive healthcare because they work with patients during vulnerable life stages. Another recommendation from climate change communications is to create support for policies without changing underlying attitudes [59]. In the case of EDCs, we expect there may be pre-existing support for some policies given that most people believe that U.S. chemicals regulation is already protective of public health.

## Conclusions

EDCs are a pervasive and invisible health threat. They are widely used in everyday products, linger in homes, and contaminate food, water, and air. They cost the U.S. economy billions of dollars in health care and lost wages [14]. As cancer rates rise among younger adults [62], fertility declines [63], and childhood behavioral disorders are common [64, 65], the health consequences of EDCs are an important communications priority. In this study, we identified expert views on the critical components of knowledge needed to make decisions about EDCs and then compared knowledge among U.S. residents with the expert assessment. Experts highlighted how EDCs disturb the body’s regulatory balance, contributing to numerous adverse health outcomes, and that evidence of harm warrants limiting exposures. They identified both personal and policy strategies for exposure reduction and cautioned that individuals need to know that current U.S. chemicals regulation does not proactively protect people from EDC exposure.

In a national survey, we observed major knowledge gaps that prevent people from making informed choices for themselves and their communities. While most respondents were aware that EDCs can affect health, they lacked knowledge about sources and exposure pathways that could inform personal action to lower exposure. Additionally, we found widespread misconceptions about U.S. chemicals regulation, including about how chemicals in products are tested for safety, and these mistaken beliefs can affect people’s perception of the need for action. Future communications that target these misconceptions in the general public have the potential to spur both individual and systems-level change towards reduced EDC exposures and harmful chemical exposures more broadly.

## Supplementary Information

Supplementary Material 1.

## Data Availability

The quantitative survey dataset collected and analyzed in this study is available in the figshare repository (DOI: 10.6084/m9.figshare.30511328). Focus group transcripts are not publicly available and may be archived at the Schlesinger Library at Harvard University where researchers may apply to access these records.

## Abbreviations

BCERP: Breast Cancer and the Environment Research Program
BPA: Bisphenol A
DDT: Dichlorodiphenyltrichloroethane
EDC: Endocrine disrupting chemical
EDC-EHL: Environmental health literacy about endocrine disruptors
EHL: Environmental health literacy
MCL: Maximum contaminant level
PBDE: Polybrominated diphenyl ether
PCB: Polychlorinated biphenyl
PEHSU: Pediatric Environmental Health Specialty Unit
PEPH: Partnerships for Environmental Public Health
PFAS: Per- and polyfluoroalkyl substances
