# Supplementary material for: What do people need to know about endocrine disrupting chemicals and health? A mental models approach using focus groups of community-engaged research teams and a national survey
Source: BMC Public Health. 2025 Nov 22;25:4414. doi: 10.1186/s12889-025-25561-4 (PMC12755022; doi:10.1186/s12889-025-25561-4)
Supplement: Supplementary file 1 — Supplementary Material 1. [file 12889_2025_25561_MOESM1_ESM.docx]

**Supplemental Material for**

What do people need to know about endocrine disrupting chemicals and health? A mental models approach using focus groups of community-engaged research teams and a national survey

Katherine E. Boronow^1*^ and Julia G. Brody^1^

^1^Silent Spring Institute, Newton, MA, USA

*Corresponding author: [boronow@silentspring.org](mailto:boronow@silentspring.org), 320 Nevada Street, Suite 302, Newton, MA 02460

**This file includes:**

Focus group moderator guide

Survey questions

Tables S1 to S3

**Focus Group Moderator Guide**

*[Note: Moderator will tailor the discussion guide to reflect the contributions and interests of the participants.]*

1. **Focus Group Preamble [3-5 minutes]**

Hello, my name is ______________, and I will be moderating this focus group today. I want to welcome you to this discussion.

Our discussion will last about 90 minutes. The goal of today’s discussion is to develop an understanding of what people need to know about endocrine disrupting chemicals, or EDCs, to make informed choices about their own health and to participate in civic debates. Everyone here has expertise on EDCs, coming from different perspectives. Thank you for being here, so we can learn from your experiences.

Our goal is to distill your knowledge down to the basic framework for what people need to know to have a functional, or action-oriented, understanding of EDC exposure and health effects. We aren’t trying to create an expert model that captures the full complexity of this topic.

I am here to foster an open discussion and to listen to your thoughts about our topic, but not to insert my own opinions. We are very interested in your ideas and want to encourage everyone to share their opinions. If your opinion is different than someone else’s, or you are unsure, still speak up, please. Everyone should be comfortable sharing ideas. Please be respectful and don’t interrupt each other. The proceedings of this focus group are private. Please do not repeat what was said here to others outside the focus group.

As you know, we will be recording this discussion. We will draw from this discussion and others like it to develop a consensus model for functional understanding of EDC exposure. We will share a summary of the focus group results with you and seek your feedback on the draft model. Your personal information will not be shared or connected to anything that you say here today.

Let’s get started.

1. **Context [10 minutes]**

The first building block to understanding endocrine disrupting chemicals, or EDCs, is being able to define them. For today’s discussion, we will consider EDCs to be “chemicals that interfere with the body’s hormone signaling system, for example, by mimicking or blocking natural hormones.”

1. What language have you found helpful in defining EDCs to lay people, whether that’s through your job or in social or family situations?
2. Do you think it’s important for people to know what EDCs are and how EDCs might affect them?
3. **Sources of exposure [15 minutes]**

We’re going to move on from considering EDCs as a general class to thinking about the specific chemicals that people might encounter in their daily lives.

1. What kinds of EDCs should people be concerned about? Please name them if you can. [KEEP LIST OF NAMED CHEMICALS/GROUPS]

- [PROMPT AS NEEDED] Should people be concerned about:
  - PFAS chemicals
  - Flame retardants
  - Parabens
  - Phthalates
  - Phenols (e.g., bisphenols, triclosan)
  - Phytoestrogens
  - [Others]

1. What do you think are the most important sources of exposure to these chemicals? [KEEP LIST OF SOURCES]

- [PROMPT AS NEEDED] Should people be concerned about EDCs in:
  - Food or food packaging
  - Personal care products
  - Cleaning products
  - Other household products
  - Drinking water
  - Pesticides
  - Industrial chemicals
  - [Others]

1. How would you say EDCs are regulated by the government? In which types of products? By what agencies?
2. **Biology of exposure [15 minutes]**

We just went over some of the sources and products that contain EDCs. Now we’re going to talk about how EDCs can get into people, and what happens inside a person’s body.

1. What are the pathways through which EDCs can enter a person’s body?

- [PROMPT AS NEEDED]
  - What about babies? Or children?
  - Are there groups of people who are at greater risk of exposure?

1. Using non-technical language, how would you describe to a friend or family member how EDCs interact with their body? We’re focusing on the basic mechanisms of EDCs right now. We’ll talk about health outcomes associated with EDCs in just a few minutes.

- [PROMPT AS NEEDED]
  - How do EDCs move through the body?
  - How do EDCs “talk” to cells and organs?
  - How does the body break down or eliminate EDCs? How long does it take?

1. **Health effects of exposure [25 minutes]**

Now we’re going to move on to the impacts of EDCs on a person’s health.

1. What kinds of health outcomes are associated with exposure to EDCs? [KEEP LIST OF HEALTH OUTCOMES]

- [PROMPT AS NEEDED] Do you think that EDC exposures can increase the risk of:
  - Breast cancer, or other reproductive cancers
  - Non-reproductive cancers
  - Fertility
  - Timing of development (puberty)
  - Brain development
  - Obesity
  - [Others]

1. What level of exposure poses a health risk?

- [PROMPT AS NEEDED]
  - Do “everyday” and “high” exposures have different health risks?
  - How would you define a “high” exposure?
  - Do “safe doses” exist?
  - Do mixtures of EDCs pose different health risks than a single EDC?

1. Are there times when people are more vulnerable to health effects from EDC exposures? When might these times be?
2. What is the strength of the evidence that EDC exposure can impact health?

- [PROMPT AS NEEDED]
  - What would you tell a friend or family member who is skeptical that EDC exposures can affect their health?

1. **Reducing exposure [15 minutes]**

The last major topic for today is how people can—or cannot—limit personal exposure to EDCs. The availability of EDC-containing products reflects a number of competing interests: manufacturers produce them, businesses sell them, consumers purchase them, and the government has the power to regulate them. Let’s discuss what individuals can do to influence each part of this equation.

1. What can individuals can do to decrease exposure to EDCs?

- [PROMPT AS NEEDED]
  - What are limits to individual purchasing decisions?
  - Can consumers pressure manufacturers and businesses?
  - Can individuals influence policy change?

1. **Wrap-up [10 minutes]**

A major challenge in communicating about EDCs is that people often hold pre-existing misconceptions. We’ll wrap up by naming some of these misconceptions, and then discussing what key information or understanding is required to “debunk” the misconception.

1. What are some common misconceptions about EDCs? [KEEP LIST]

- [FOR EACH MISCONCEPTION, PROMPT]
  - How would you explain to a friend or family member why that isn’t true?

1. Is there anything that hasn’t been mentioned yet that people should know about EDCs and health?

**Thank you for your participation!**

**Survey questions**

The next questions are about chemicals in everyday consumer products. For each statement, tell us if you think it is true, probably true, probably false, or false. We want to know what you think is the best answer. It’s OK to make your best guess.

*[Questions in this section were randomized. Answers were repeated for each question.]*

1. Everyday products—like shampoo, sofas, and plastic bottles—sometimes contain chemicals that can upset the balance of hormones in a person’s body. (T)
   1. True
   2. Probably true
   3. Probably false
   4. False
2. Tests by the U.S. Centers for Disease Control and Prevention (CDC) show that everyone has chemicals from the environment detected in their body.* (T)
3. Wiping dusty surfaces with a damp cloth is a good way to remove harmful chemicals from your home. (T)
4. Washing your hands has no effect on your exposure to harmful chemicals.* (F)
5. Eating unpackaged fresh foods can reduce your exposure to chemicals, even if the food is not organic. (T)
6. A product labeled “BPA-free” will not contain any toxic chemicals. (F)
7. Some stores and online companies won’t sell products that contain certain harmful chemicals. (T)
8. Chemicals have to pass many safety tests before they can be used in products in the U.S.* (F)
9. In the U.S., companies aren’t allowed to use chemicals that are similar to those known to be harmful. (F)
10. Companies are required to tell consumers all the chemicals they put in household goods like paint, pots and pans, or carpet. (F)
11. Reading the label will tell you all of the chemicals in household cleaning products. (F)
12. Banning certain chemicals from consumer products has led to lower levels of those chemicals in Americans. (T)
13. Chemicals that affect hormones are not found in public drinking water. (F)
14. Babies in the womb are not exposed to chemicals from household products before they are born.* (F)
15. Chemicals used in food packaging do not get into the food. (F)
16. Your skin stops chemicals in lotions and makeup from entering your body. (F)
17. People can get chemicals in their blood from the dust in their home.* (T)
18. The chemicals inside furniture and electronics stay inside them, so people can’t get exposed to chemicals from those things. (F)
19. Small amounts of hormone-like chemicals won’t affect your body. (F)
20. Many small exposures to chemicals can add up to pose a health risk. (T)
21. Chemical exposures that happened years ago do not affect a person’s current health. (F)
22. A baby’s exposure to chemicals can increase their risk for some diseases as an adult. (T)
23. A woman’s chemical exposures during pregnancy will not affect her grandchildren’s health. (F)
24. Some chemicals can remain in a person’s body for years.* (T)
25. Hormones are your body’s chemical messengers that travel in your blood and give instructions to different parts of the body. (T)
26. Some chemicals can upset the balance of hormones in your body. (T)
27. Nearly all of your body’s systems are controlled by hormones. (T)
28. Chemicals that affect hormones can make cancer grow faster or slower. (T)
29. More than half of your cancer risk is inherited in your genes. (F)
30. Some chemicals in everyday products can affect the ability of a person to have children.* (T)
31. Exposure in early life to certain chemicals that affect hormones can affect a baby’s brain development and IQ.* (T)
32. The American Academy of Pediatrics recommends fresh or frozen fruits and vegetables because of concerns about chemicals in food packaging. (T)
33. Scientists are not sure yet about all the health implications of chemicals that can upset a person’s natural balance of hormones.* (T)
34. A doctor can tell if you will get sick based on the levels of chemicals in your body.* (F)

*[*Starred questions were adapted from:* MyCHDS Report Study, reported in Boronow KE, Cohn B, Havas L, Plumb M, Brody JG. The Effect of Individual or Study-Wide Report-Back on Knowledge, Concern, and Exposure-Reducing Behaviors Related to Endocrine-Disrupting Chemicals. Environmental Health Perspectives. 2023;131(9):097005.*]*

Below is a list of common items that you might find in someone’s home. For each item, tell us if you think it is likely to contain chemicals that affect people’s hormones.

*[Questions in this section were randomized.]*

| Is the item likely to contain chemicals that affect people’s hormones? | No | Yes | I don’t know |
| --- | --- | --- | --- |
| 1. Microwave popcorn |  | (X) |  |
| 1. Canned food |  | (X) |  |
| 1. Cast iron frying pan | (X) |  |  |
| 1. Plastic food storage containers |  | (X) |  |
| 1. Chemical sunscreen (non-mineral) |  | (X) |  |
| 1. Vinegar cleaning solution | (X) |  |  |
| 1. Air freshener |  | (X) |  |
| 1. Glass water bottle | (X) |  |  |
| 1. Baking soda | (X) |  |  |
| 1. Stainless steel straw | (X) |  |  |

You may have heard about some chemicals, for example, on product packaging, in news stories, or in talking to others. For each chemical, tell us whether you have heard of it before. Then, choose the statement that is true about the chemical.

1. Have you heard of PFAS (per- and polyfluoroalkyl substances) before?
   1. No
   2. Yes
      1. [IF YES] Which of these statements is true for PFAS (per- and polyfluoroalkyl substances)?
         1. PFAS are added to furniture and electronics to keep them from catching on fire.
         2. PFAS are used to make things non-stick, waterproof, or grease-resistant. (T)
         3. PFAS are added to cosmetics to keep them from spoiling.
         4. PFAS are used in polycarbonate plastic, receipt paper, and canned food linings.
         5. I don’t know
2. Have you heard of BPA (bisphenol A) before?
   1. No
   2. Yes
      1. [IF YES] Which of these statements is true for BPA (bisphenol A)?
         1. BPA is added to furniture and electronics to keep them from catching on fire.
         2. BPA is used to make things non-stick, waterproof, or grease-resistant.
         3. BPA is added to cosmetics to keep them from spoiling.
         4. BPA is used in polycarbonate plastic, receipt paper, and canned food linings. (T)
         5. I don’t know
3. Have you heard of parabens before?
   1. No
   2. Yes
      1. [IF YES] Which of these statements is true for parabens?
         1. Parabens are added to furniture and electronics to keep them from catching on fire.
         2. Parabens are used to make things non-stick, waterproof, or grease-resistant.
         3. Parabens are added to cosmetics to keep them from spoiling. (T)
         4. Parabens are used in polycarbonate plastic, receipt paper, and canned food linings.
         5. I don’t know

**Table S1.** Response frequencies for the true-false knowledge questions (n = 504). Questions are grouped by mental model domain and ordered by descending percent correct.

|  | | | **Number (%)** | | | |
| --- | --- | --- | --- | --- | --- | --- |
| **Question** | **Correct Answer** | **Domain** | **Correct** | **Probably correct** | **Probably incorrect** | **Incorrect** |
| Some chemicals can upset the balance of hormones in your body. | True | Biology | 268 (53.2) | 199 (39.5) | 32 (6.3) | 5 (1) |
| Some chemicals can remain in a person's body for years. | True | Biology | 282 (56) | 184 (36.5) | 33 (6.5) | 5 (1) |
| Many small exposures to chemicals can add up to pose a health risk. | True | Biology | 253 (50.2) | 208 (41.3) | 40 (7.9) | 3 (0.6) |
| A baby's exposure to chemicals can increase their risk for some diseases as an adult. | True | Biology | 229 (45.4) | 230 (45.6) | 37 (7.3) | 8 (1.6) |
| Exposure in early life to certain chemicals that affect hormones can affect a baby's brain development and IQ. | True | Biology | 210 (41.7) | 242 (48) | 44 (8.7) | 8 (1.6) |
| Chemicals that affect hormones can make cancer grow faster or slower. | True | Biology | 168 (33.3) | 282 (56) | 45 (8.9) | 9 (1.8) |
| Hormones are your body's chemical messengers that travel in your blood and give instructions to different parts of the body. | True | Biology | 212 (42.1) | 221 (43.8) | 57 (11.3) | 14 (2.8) |
| Some chemicals in everyday products can affect the ability of a person to have children. | True | Biology | 173 (34.3) | 253 (50.2) | 70 (13.9) | 8 (1.6) |
| Chemical exposures that happened years ago do not affect a person's current health. | False | Biology | 250 (49.6) | 165 (32.7) | 63 (12.5) | 26 (5.2) |
| Nearly all of your body's systems are controlled by hormones. | True | Biology | 144 (28.6) | 242 (48) | 95 (18.8) | 23 (4.6) |
| Small amounts of hormone-like chemicals won't affect your body. | False | Biology | 105 (20.8) | 225 (44.6) | 147 (29.2) | 27 (5.4) |
| A woman's chemical exposures during pregnancy will not affect her grandchildren's health. | False | Biology | 134 (26.6) | 195 (38.7) | 127 (25.2) | 48 (9.5) |
| More than half of your cancer risk is inherited in your genes. | False | Biology | 35 (6.9) | 131 (26) | 263 (52.2) | 75 (14.9) |
| Everyday products - like shampoo, sofas, and plastic bottles - sometimes contain chemicals that can upset the balance of hormones in a person's body. | True | Exposure | 177 (35.1) | 258 (51.2) | 51 (10.1) | 18 (3.6) |
| Babies in the womb are not exposed to chemicals from household products before they are born. | False | Exposure | 199 (39.5) | 197 (39.1) | 76 (15.1) | 32 (6.3) |
| Chemicals used in food packaging do not get into the food. | False | Exposure | 161 (31.9) | 235 (46.6) | 76 (15.1) | 32 (6.3) |
| Your skin stops chemicals in lotions and makeup from entering your body. | False | Exposure | 208 (41.3) | 179 (35.5) | 84 (16.7) | 33 (6.5) |
| Chemicals that affect hormones are not found in public drinking water. | False | Exposure | 120 (23.8) | 229 (45.4) | 117 (23.2) | 38 (7.5) |
| The chemicals inside furniture and electronics stay inside them, so people can't get exposed to chemicals from those things. | False | Exposure | 137 (27.2) | 211 (41.9) | 112 (22.2) | 44 (8.7) |
| People can get chemicals in their blood from the dust in their home. | True | Exposure | 121 (24) | 219 (43.5) | 127 (25.2) | 37 (7.3) |
| Washing your hands has no effect on your exposure to harmful chemicals. | False | Exposure | 155 (30.8) | 183 (36.3) | 117 (23.2) | 49 (9.7) |
| Wiping dusty surfaces with a damp cloth is a good way to remove harmful chemicals from your home. | True | Exposure | 105 (20.8) | 193 (38.3) | 147 (29.2) | 59 (11.7) |
| Eating unpackaged fresh foods can reduce your exposure to chemicals, even if the food is not organic | True | Exposure | 66 (13.1) | 229 (45.4) | 156 (31) | 53 (10.5) |
| Tests by the U.S. Centers for Disease Control and Prevention (CDC) show that everyone has chemicals from the environment detected in their body. | True | Society | 185 (36.7) | 262 (52) | 46 (9.1) | 11 (2.2) |
| Banning certain chemicals from consumer products has led to lower levels of those chemicals in Americans. | True | Society | 155 (30.8) | 285 (56.5) | 54 (10.7) | 10 (2) |
| Some stores and online companies won't sell products that contain certain harmful chemicals. | True | Society | 196 (38.9) | 208 (41.3) | 71 (14.1) | 29 (5.8) |
| A product labeled "BPA-free" will not contain any toxic chemicals. | False | Society | 66 (13.1) | 172 (34.1) | 200 (39.7) | 66 (13.1) |
| Reading the label will tell you all of the chemicals in household cleaning products. | False | Society | 48 (9.5) | 154 (30.6) | 185 (36.7) | 117 (23.2) |
| In the U.S., companies aren't allowed to use chemicals that are similar to those known to be harmful. | False | Society | 46 (9.1) | 141 (28) | 205 (40.7) | 112 (22.2) |
| Companies are required to tell consumers all the chemicals they put in household goods like paint, pots and pans, or carpet. | False | Society | 25 (5) | 111 (22) | 182 (36.1) | 186 (36.9) |
| Chemicals have to pass many safety tests before they can be used in products in the U.S. | False | Society | 13 (2.6) | 77 (15.3) | 239 (47.4) | 175 (34.7) |
| Scientists are not sure yet about all the health implications of chemicals that can upset a person's natural balance of hormones. | True | Uncertainty | 193 (38.3) | 233 (46.2) | 57 (11.3) | 21 (4.2) |
| The American Academy of Pediatrics recommends fresh or frozen fruits and vegetables because of concerns about chemicals in food packaging. | True | Uncertainty | 148 (29.4) | 269 (53.4) | 75 (14.9) | 12 (2.4) |
| A doctor can tell if you will get sick based on the levels of chemicals in your body. | False | Uncertainty | 29 (5.8) | 145 (28.8) | 240 (47.6) | 90 (17.9) |

**Table S2.** Response frequencies for the household item questions (n = 504). Questions are grouped by source type (likely or not likely to contain EDCs) and ordered by descending percent correct.

|  | | **Number (%)** | | |
| --- | --- | --- | --- | --- |
| **Household item** | **Correct Answer** | **Correct** | **Incorrect** | **Don't know** |
| Glass water bottle | Not likely | 304 (60.3) | 117 (23.2) | 83 (16.5) |
| Baking soda | Not likely | 288 (57.1) | 116 (23) | 100 (19.8) |
| Vinegar cleaning solution | Not likely | 277 (55) | 137 (27.2) | 90 (17.9) |
| Stainless steel straw | Not likely | 230 (45.6) | 150 (29.8) | 124 (24.6) |
| Cast iron frying pan | Not likely | 220 (43.7) | 180 (35.7) | 104 (20.6) |
| Chemical sunscreen (non-mineral) | Likely | 367 (72.8) | 53 (10.5) | 84 (16.7) |
| Air freshener | Likely | 362 (71.8) | 67 (13.3) | 75 (14.9) |
| Plastic food storage containers | Likely | 362 (71.8) | 84 (16.7) | 58 (11.5) |
| Canned food | Likely | 282 (56) | 126 (25) | 96 (19) |
| Microwave popcorn | Likely | 255 (50.6) | 153 (30.4) | 96 (19) |

**Table S3.** Response frequencies for the chemical group source questions, among participants who responded that they had heard of the chemical before.

| **Chemical group** | **Source statement** | **Graded response** | **Number (%)** |
| --- | --- | --- | --- |
| BPA (n=270) | BPA is used to make things non-stick, waterproof, or grease-resistant. | Incorrect | 37 (13.7) |
|  | BPA is used in polycarbonate plastic, receipt paper, and canned food linings. | Correct | 126 (46.7) |
|  | BPA is added to furniture and electronics to keep them from catching on fire. | Incorrect | 9 (3.3) |
|  | BPA is added to cosmetics to keep them from spoiling. | Incorrect | 6 (2.2) |
|  | No response | No response | 92 (34.1) |
| PFAS (n=85) | PFAS are used to make things non-stick, waterproof, or grease-resistant. | Correct | 32 (37.6) |
|  | PFAS are used in polycarbonate plastic, receipt paper, and canned food linings. | Incorrect | 12 (14.1) |
|  | PFAS are added to furniture and electronics to keep them from catching on fire. | Incorrect | 14 (16.5) |
|  | PFAS are added to cosmetics to keep them from spoiling. | Incorrect | 2 (2.4) |
|  | No response | No response | 25 (29.4) |
| Parabens (n=281) | Parabens are used to make things non-stick, waterproof, or grease-resistant. | Incorrect | 33 (11.7) |
|  | Parabens are used in polycarbonate plastic, receipt paper, and canned food linings. | Incorrect | 31 (11) |
|  | Parabens are added to furniture and electronics to keep them from catching on fire. | Incorrect | 11 (3.9) |
|  | Parabens are added to cosmetics to keep them from spoiling. | Correct | 122 (43.4) |
|  | No response | No response | 84 (29.9) |
